# Characterization of *B-box* family genes and their expression profiles under abiotic stresses in the *Melilotus albus*

**DOI:** 10.3389/fpls.2022.990929

**Published:** 2022-09-29

**Authors:** Lili Nian, Xiaoning Zhang, Xingyu Liu, Xiaodan Li, Xuelu Liu, Yingbo Yang, Fasih Ullah Haider, Xiaolin Zhu, Biao Ma, Zixuan Mao, Zongyang Xue

**Affiliations:** ^1^College of Forestry, Gansu Agricultural University, Lanzhou, China; ^2^College of Management, Gansu Agricultural University, Lanzhou, China; ^3^College of Resources and Environmental Sciences, Gansu Agricultural University, Lanzhou, China; ^4^College of Agronomy, Gansu Agricultural University, Lanzhou, China

**Keywords:** B-box, *Melilotus albus*, phylogenetic analysis, gene expression, abiotic stresses

## Abstract

B-box (BBX) proteins are one of the zinc-finger transcription factor that plays a critical role in plant development, growth, and multiple stress responses. Although *BBX* genes have been reported in many model organisms, no comprehensive study has yet been conducted on the BBX genes in *Melilotus albus*, and the biological functions of this family remain unknown. In this study, a total of 20 *BBX* (MaBBX) genes were identified in *M*. *albus* and were phylogenetically divided into five clades. *BBX* members within the same clade showed similar conserved domain, suggesting similarity of potential biological function. Analysis of MaBBX conserved motifs showed that every subfamily contained two common motifs. Distribution mapping shows that BBX proteins are nonrandomly localized in eight chromosomes. The synteny showed that most homologous gene pairs of the MaBBX gene family were amplified by segmental replication, which meant segmental replication was the main way for the MaBBX gene family to evolve. Additionally, the *cis*-element analysis predicted light-responsive, various hormone and stress-related elements in the promoter regions of MaBBXs. Furthermore, the expression levels of all 20 MaBBX genes were detected by qRT-PCR under salt, cold, and dark stresses in *M. albus*. Moreover, it was observed that 16 genes had higher expression levels after 3 h of salt treatment, 10 genes were significantly upregulated after 3 h of cold treatment, and all genes were up regulated after 3 h of dark treatment, and then appeared to decline. In addition, it was also noticed that *MaBBX13* may be an important candidate for improving tolerance to abiotic stress. The prediction of protein tertiary structure showed that the tertiary structures of members of the same subfamily of MaBBX proteins were highly similar. The hypothesis exhibited that most of the MaBBX proteins were predicted to be localized to the nucleus and cytoplasm and was validated by transient expression assays of MaBBX15 in tobacco leaf epidermal cells. This study provides useful information for further investigating and researching the regulatory mechanisms of *BBX* family genes in response to abiotic stresses in *M. albus*.

## Background

Transcription factors (TFs) are important regulatory factors in organisms and are widely involved in the regulation of plant growth, development, metabolism, and environmental stress response. Zinc finger proteins (ZFPs) are a class of TFs with a zinc finger structure composed of histidine (His), cysteine (Cys), and zinc ions. The protein family is divided into several subfamilies according to the differences in the conserved domains of zinc finger structure transcription factors (Riechmann et al., 2000; Laity et al., 2001). BBX (B-box) is a subfamily of the zinc finger structure transcription factor family, its amino acid sequence contains one or two B-box domains at the N-terminus, or an additional CCT (CONSTANS, CO-like, and TOC1) domain or VP motif at the C-terminus (Gangappa and Botto, 2014).

The B-box gene was first discovered in a late-flowering mutant of *Arabidopsis thaliana*, named *CONSTANS* (*CO*), and it was confirmed that the gene is involved in the regulation of plant flowering and other life activities (Putterill et al., 1995). Subsequent studies further revealed that plant B-box transcription factors are involved in the regulation of seed germination (Chang et al., 2011), flowering (González-Schain et al., 2012), and shade avoidance response (Crocco et al., 2011). Furthermore, it plays an important role in life activities such as biotic or abiotic stress response (Wang et al., 2013a) and plant hormone signal transduction (Fan et al., 2012). For example, the *Arabidopsis* AtBBX1 protein is a core factor regulating the flowering pathway under long-day conditions (Putterill et al., 1995). AtBBX21 is a positive regulator of photomorphogenesis that directly binds to the T/G-box motif in the HY5 promoter and activates its expression (Xu et al., 2018). AtBBX25 can form an inactive heterodimer with HY5, which negatively regulates the expression of AtBBX22. This process inhibits photomorphogenesis in seedlings (Gangappa et al., 2013). AtBBX5 participates in the response to abiotic stress through an abscisic acid (ABA) dependent signaling pathway and is an important regulator of plant tolerance to abiotic stress (Min et al., 2015). The expression of *COL* (*CONSTANSlike*) genes in the *BBX* gene family of rice (*Oryza sativa*) was lower under drought and high-temperature stress, but significantly increased under low-temperature stress (Weng et al., 2014). The overexpression of *AtBBX18* in *Arabidopsis thaliana* can reduce the heat tolerance of plants (Wang et al., 2013b). *MdMYB1* and *MdMYB9* are two key positive regulators of anthocyanin biosynthesis in apples (*Malus domestica*), recent studies have shown that MdBBX37 interacts with MdMYB1 and MdMYB9 to inhibit the binding of these two proteins to their target genes, thereby negatively regulating anthocyanins biosynthesis (Bai et al., 2014; An et al., 2020). In a previous study (Huang et al., 2012), rice seedlings at the germ and radicle stage were exposed to 48 h of continuous light or darkness. Compared with dark conditions, all eight studied genes were upregulated under light conditions. In summary, *BBX* genes may have irreplaceable effects on various biological processes, but little is known about the *BBXs* in *Melilotus albus*.

*Melilotus albus* has a large yield, strong stress resistance, strong nitrogen fixation ability, and high nutritional value. *Melilotus albus* is an excellent forage grass, which can provide a large amount of feed for animal husbandry. In the mid-15th century, *M. albus* has been used in medicine to treat ophthalmia, hemorrhoids, phlebitis, varicose veins, and other diseases (Pacanoski, 2010). *Melilotus albus* contains essential oils and flavonoids, such as coumarin glycosides, which give *M. albus* a special aromatic odor, so it has moisturizing and carminative properties. It is used medicinally as an emollient and digestive aid and is recommended for many diseases (Yang et al., 2014a). However, with the increasingly severe global climate and environmental situation, the growth of *M. albus* has been seriously threatened. The study on the functions and regulatory mechanism of the genes related to abiotic stress response in *M. albus* is of great significance to the stress-resistant molecular-assisted breeding of *M. albus*. At present, the genome sequencing of *M. albus* has been completed (Wu et al., 2022), which lays the foundation for the systematic evolution and functional analysis of the *BBX* gene family of *M. albus* utilizing bioinformatics. In this study, for the first time, we systematically identified the *MaBBXs* at the genome level in the *M. albus* and performed their exon-intron structure analysis. Moreover, we also examined expression patterns of *MaBBXs* under various abiotic stress treatments. The current study provided the foundation for further investigating the function of *BBX* genes in *M. albus* growth and development, and resistance to abiotic stress.

## Materials and methods

### Identification of BBX family members in *Melilotus albus*

B-box protein sequences of *Arabidopsis* and rice were downloaded from (TAIR10, http://www.arabidopsis.org/index.jsp) and (TIGR, http://rice.plantbiology.msu.edu/index.shtml) respectively. *Arabidopsis* contains 32 and rice contains 30 BBX proteins (Ouyang et al., 2007; Berardini et al., 2015; Supplementary Table 1). The genome sequence data of M. albus were obtained from NCBI (Wu et al., 2022). First, all the 32 *Arabidopsis* BBX proteins and the 47 rice BBX proteins were used as queries for the BLAST program (*e*-value <0.00001; Camacho et al., 2009) to identify *BBX* homologs in *M. albus*. Then, the Inter Pro database (Apweiler et al., 2001) and NCBI’s CDD tool (Marchler-Bauer et al., 2017) were used to determine the domains of candidate proteins. The protein sequences without the B-box domain were removed, and finally, all members of the *M. albus BBX* gene family were obtained. The Protparam tool provided by the ExPASy online website (https://www.expasy.org/; Gasteiger et al., 2003) was used to analyze the structural properties of the amino acid sequence of MaBBX protein, thereby obtaining the physicochemical properties such as amino acid length, molecular weight, isoelectric point, and hydrophobicity of MaBBX protein. Subcellular localization of MaBBX proteins was performed by the online tools Psort-Prediction^1^ and Cell-PLoc2.0^2^ prediction.

### Chromosomal location, patterns of gene duplication, and synteny analysis

The chromosomal location information of all *MaBBX* gene family members was extracted using TBtools (Chen et al., 2020), and the chromosomal location distribution of *MaBBX* was visualized using Map Charts 2.2. *MaBBX* genes were named according to the position in which they appear on the chromosome. Syntenic blocks for the *M. albus BBX* genes were identified and analyzed using the MCScanX in TBtools. Synteny analysis and chromosomal location diagrams were generated in a globe plot using the program Circos (http://circos.ca, Accessed on November 12, 2021) in the TBtools. Conditions for determining gene duplication events: (1) The matching length of the two gene sequences was greater than 80% of the length of the longer sequence; (2) The similarity of the matching parts of the two gene sequences was greater than 80%; and (3) Among closely linked genes, participated in only one replication event (Jiang et al., 2013; Kong et al., 2013). Further combined with the location of the *BBX* gene on the chromosome, it was determined whether it was tandem replication or Segmental replication. The nonsynonymous (Ka) and synonymous (Ks) substitution rates of each gene pair were calculated using the TB-tools software. For the timing of duplication events, the formula: T = Ks/2λ × 10^−6^ Mya was used to calculate divergence time (T) in millions of years (Mya), where λ = 4.5 × 10^−9^ represented the evolution rate of *M. albus.*

### Phylogenetic analysis

A protein sequence file (Supplementary Table 1) containing all the *BBX* family members of *M. albus*, *A. thaliana*, and rice was compiled by the TBtools, and the amino acid sequences were aligned by the MUSCLE tool in the MEGA7.0 software (Kumar et al., 2018). According to the comparison results, the best amino acid substitution model was selected by the model selection tool MEGA7.0. Second, in MEGA7.0, a phylogenetic tree containing amino acid sequences of *M. albus*, *Arabidopsis*, and rice was constructed by the maximum likelihood. For statistical reliability, the nodes of the tree were assessed by bootstrap analysis containing 1,000 replicates. Finally, the phylogenetic tree was further visualized and beautified by the iTOL online tool.^3^

### Analysis of the conserved motifs and gene structures

According to the GFF annotation file information of the genome of *M. albus*, the online website Gene Structure Display Server (GSDS; http://gsds.gao-lab.org/; Guo et al., 2007) was used to obtain the visualization of exon-intron. The online tool MEME (https://meme-suite.org/meme/; Bailey et al., 2009) was used to predict the conserved motifs present in the *BBX* gene, and the parameters were set as follows: the number of predicted motifs was 5, and the motif length was 6–50 amino acids (Supplementary Table 3). At the same time, the TBtools software was used to draw the gene evolution tree, gene structure, and motif comprehensive map of the *BBX* gene family of *M. albus*.

### Analysis of cis-acting elements of MaBBX genes, MaBBXs protein–protein interaction networks, and prediction of protein tertiary structure

To analyze the *cis*-acting elements in the promoter region, the DNA sequence 2000 bp upstream of the initiation codon was obtained from the genome sequence of *M. albus*, and then the obtained sequence was submitted to Plant CARE (http://bioinformatics.psb.ugent.be/webtools/plantcare/html/; Rombauts et al., 1999) database to finally identify possible *cis*-acting elements in the promoter region (Supplementary Table 4). STRING 10 (http://string-db.org/; Szklarczyk et al., 2015) was used to construct an interaction network, with interolog proteins from *Arabidopsis*, to analyze the relationship of MaBBXs with other proteins. At the same time, we used the SWISS-MODEL (Biasini et al., 2014; https://swissmodel.expasy.org/interactive) provided by the online software ExPaSy to establish the tertiary structure model of the *MaBBX* gene family of *M. albus*. Ramachandran plot is a visualization method that reflects whether the conformation of protein is reasonable (Laskowski et al., 1993). The online site PDBsum^4^ was used to complete the Ramachandran plot.

### Plant material and stress treatments

The *M. albus* seeds with uniform shape and full grains were selected. The seed coat was removed from seeds and sterilized the seeds with 75% alcohol for 3–5 min, and then rinsed with sterile deionized water (4–5 times). Finally, they were spread in Petri dishes covered with sterile filter paper and placed in an incubator at ambient conditions of 24°C (temperature), 80% (relative humidity), and 16/8 h (day/night, photoperiod), and the light intensity of 4,000 lx. After the cotyledons were opened, the seedlings were transferred to the nutrient soil pots with a ratio of 3:2:1 of peat soil: vermiculite: and perlite for further cultivation. The 4-week-old *M. albus* plants with the same growth state were subjected to three stress treatments: (1) Salt stress: water the *M. albus* plants from the roots of 200 mmol/L NaCl, 300 ml per pot; (2) cold stress: 4°C treatments; and (3) dark stress treatments. *Melilotus albus* plants were sampled at 0, 3, 6, 9, 12, 24, and 48 h after the initiation of the treatments. The sampled tissue was the leaves of the *M. albus*. All samples were collected in triplicate, then frozen in lipid nitrogen, and finally stored at −80°C for subsequent use according to the procedure mentioned by Nian et al. (2021).

### RNA extraction, reverse transcription, and qRT-PCR analysis of *Melilotus albus*

Total RNA from each sample was extracted using UNIQ-10 Columnar Trizol Total RNA Extraction Kit (Sango Biotech). Total RNA concentration and quality were analyzed using an Ultrasec™ 2100 pro UV/ Visible spectrophotometer (Amersham Biosciences) and gel-electrophoresis. Using the M-MuLV first-strand cDNA synthesis kit (Sangon Bio), 1 μg of total RNA was used for reverse transcription of cDNA, and the cDNA samples were diluted to a concentration of 1 ng/ml for use. SYBR Green PCR Master Mix (Applied Biosystems, United Kingdom) was used for fluorescent dyes, and sample qRT-PCR validation was performed on an Applied Biosystems 7500 real-time PCR system. β-tubulin was selected as the internal reference gene (Knopkiewicz and Wojtaszek, 2019). Validation of reference genes for gene expression analysis using quantitative polymerase chain reaction in pea lines (*Pisum sativum*) with different lodging susceptibility. And Primer Premier 6.0 software was used to design specific primers for the *MaBBX* gene to be tested and the internal reference gene (Supplementary Table 5). The reaction system was: 0.4 μl of upstream and downstream primers, 1 μl of cDNA sample, 5 μl of fluorescent dye, and 3.2 μl of sterile water. The qRT-PCR reaction program was; pre-denaturation at 95°C for 5 min, denaturation at 95°C for 15 s, annealing at 60°C for 1 min, 40 cycles. In addition, this study used the 2-ΔΔCt method (Livak and Schmittgen, 2001) to calculate the relative expression levels of genes. Each experiment was repeated three times with independent RNA samples. SPSS statistical software was used to analyze the relative expression levels of each gene at different sampling points under salt, cold, and dark stress treatment. The graphical representation of the experimental findings was produced by using Graphpad. Different letters in the figures indicate that the mean values of different sampling times are significantly different, which was determined by the Tukey pairwise comparison test.

### Subcellular localization of BBX proteins

To verify the localization of *MaBBX15* in plant cells, the *MaBBX15* gene was selected to design primers with a homologous recombination arm (Supplementary Table 6), and the coding sequences of *MaBBX15* were amplified and cloned into the GFP: pCAMBIA 1302 vector. The successfully constructed plasmids were separately transformed into *Agrobacterium* strain GV3101. For transient expression, Tobacco (*Nicotiana benthamiana*) plants were cultivated in a greenhouse at 23°C for about 1 month, then the tobacco in good growth condition was selected, and *Agrobacterium tumefaciens* carrying the recombinant vector was injected into the third or fourth leaf and incubated at 25°C for 24 h in the dark, followed by 24–72 h in the light, to make sections for observation under a laser confocal microscope (Olympus, Tokyo, Japan; Wei et al., 2020).

## Results

### Identification and of *BBX* family members in *Melilotus albus*

A total of 20 *MaBBX* genes were identified. For the sake of consistency, all of the *MaBBXs* were named from *MaBBX01* to *MaBBX20* according to their relative positions on the chromosome. The detailed basic information for *MaBBX* genes were listed in Table 1, such as the gene name, gene ID, length of the protein, molecular weight (MW), theoretical isoelectric point (PI), grand average of hydropathicity (GRAVY), and subcellular localization. The protein length, MW, and pI for 20 MaBBX proteins varied widely. Further analysis revealed that their length ranged from 185 aa (MaBBX15) to 431 aa (MaBBX01), with an average length of 304 aa; the MW ranged from 20.34 kD (MaBBX15) to 48.22 kD (MaBBX01), with an average MW of 33.88 kD; and the pI ranged from 4.62 to 9.01, with an average pI of 5.88, indicating that most of these proteins were acidic in nature. The GRAVY of all MaBBX proteins is less than 0, indicating that MaBBX proteins are hydrophilic proteins. The results of subcellular localization in different online tools are inconsistent, and the prediction results of Cell-PLoc2.0 show that all MaBBX transcription factor families are distributed in the nucleus of *M. albus*. However, the prediction results of Psort-Prediction indicated that five of the 20 MaBBX transcription factor families were distributed in the nucleus, seven in the cytoplasm, and the remaining eight in the mitochondrial matrix of *M. albus*.

**Table 1:** Basic information of 20 *MaBBXs* identified in *M. albus*.

Gene Name	Gene ID	Protein	Molecular	Isoelectric	GRAVY	Subcellular Localization	
		length (aa)	weight (kDa)	point (PI)		Cell-PLoc2.0	Psort-Prediction
MaBBX01	Malbus0100335.1	431	48.22	5.22	−0.692	Nucleus	Nucleus
MaBBX02	Malbus0100420.1	344	38.99	4.99	−0.458	Nucleus	Mitochondrial matrix space
MaBBX03	Malbus0105975.1	277	30.71	6.54	−0.499	Nucleus	Mitochondrial matrix space
MaBBX04	Malbus0200283.1	271	30.02	6.81	−0.394	Nucleus	Cytoplasm
MaBBX05	Malbus0200509.1	221	24.46	5.81	−0.298	Nucleus	Cytoplasm
MaBBX06	Malbus0202130.1	411	45.31	4.99	−0.635	Nucleus	Nucleus
MaBBX07	Malbus0203858.1	244	26.75	9.01	−0.227	Nucleus	Cytoplasm
MaBBX08	Malbus0300494.1	407	45.06	5.12	−0.614	Nucleus	Mitochondrial matrix space
MaBBX09	Malbus0303046.1	219	24.73	5.19	−0.486	Nucleus	Mitochondrial matrix space
MaBBX10	Malbus0304583.1	358	40.3	6.13	−0.69	Nucleus	Cytoplasm
MaBBX11	Malbus0400450.1	243	27.44	5.03	−0.603	Nucleus	Mitochondrial matrix space
MaBBX12	Malbus0401237.1	225	25.06	4.62	−1.034	Nucleus	Mitochondrial matrix space
MaBBX13	Malbus0401677.1	295	32.41	5.04	−0.648	Nucleus	Cytoplasm
MaBBX14	Malbus0505312.1	377	41.22	5.86	−0.384	Nucleus	Cytoplasm
MaBBX15	Malbus0601060.1	185	20.34	7.01	−0.581	Nucleus	Cytoplasm
MaBBX16	Malbus0602171.1	314	35.35	6.49	−0.601	Nucleus	Mitochondrial matrix space
MaBBX17	Malbus0702427.1	241	26.73	4.81	−0.35	Nucleus	Mitochondrial matrix space
MaBBX18	Malbus0702607.1	199	22.38	8.15	−0.425	Nucleus	Nucleus
MaBBX19	Malbus0705261.1	416	47.06	5.1	−0.698	Nucleus	Nucleus
MaBBX20	Malbus0802069.1	412	45.11	5.74	−0.607	Nucleus	Nucleus

### Chromosomal location and synteny analysis of *MaBBXs*

**Figure 1:**
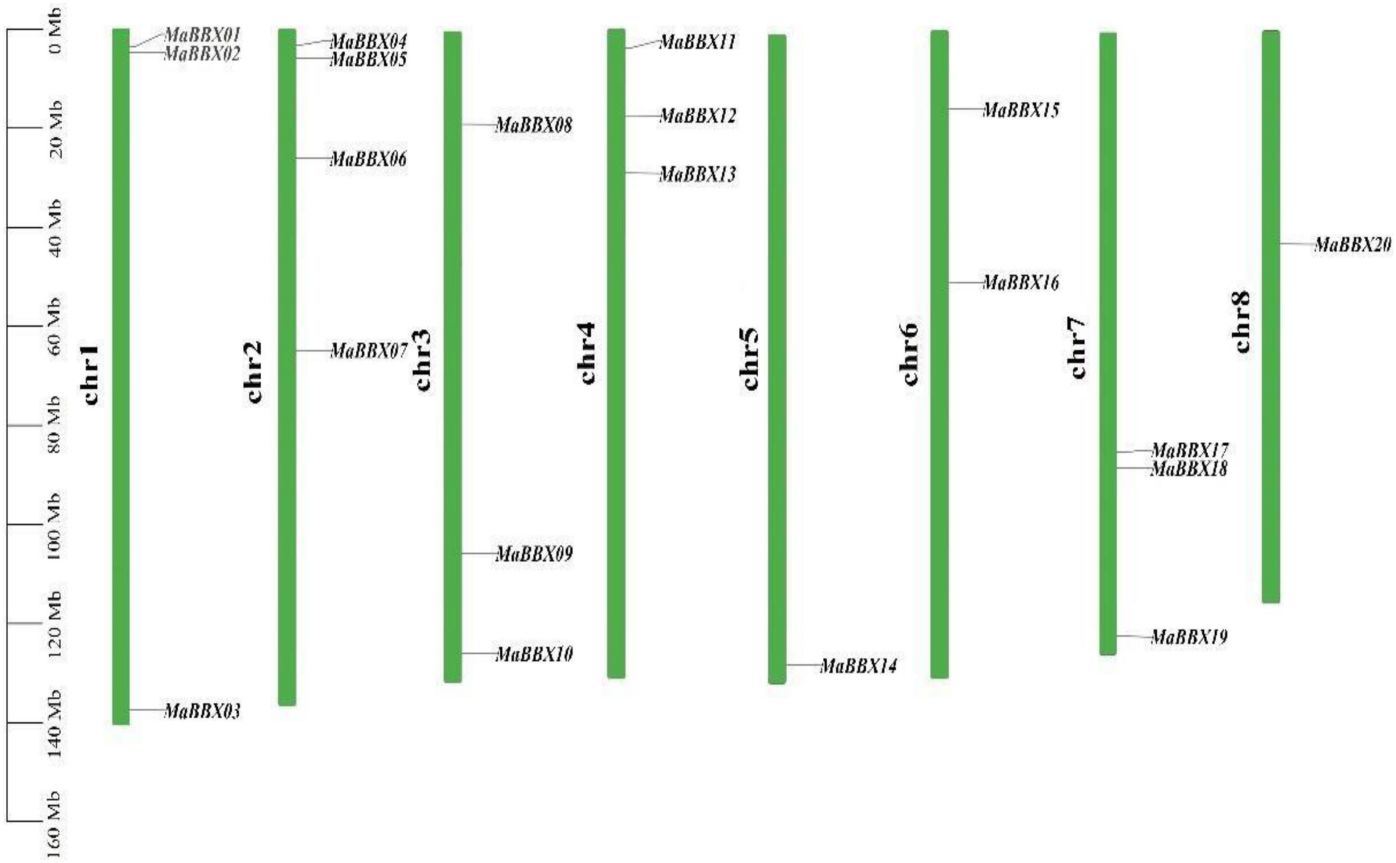
The distribution of *MaBBXs* in *Melilotus albus* chromosomes. Twenty identified *MaBBXs* were mapped to the eight chromosomes. The chromosome name is at the left of each bar.

In general, *MaBBXs* were unevenly distributed in the *M. albus* chromosomes (Figure 1). For example, one gene was distributed on the chr5 (*MaBBX14*) and chr8 (*MaBBX20*) chromosomes respectively, Chr2 has the largest number of members, with four genes (*MaBBX04*, *MaBBX05*, *MaBBX06*, and *MaBBX07*) and the other five chromosomes have 2–3 genes each. In terms of the distribution of chromosomes, *MaBBX01*, *MaBBX02*, *MaBBX04*, *MaBBX05*, *MaBBX06*, *MaBBX08*, *MaBBX11*, *MaBBX12*, *MaBBX13*, and *MaBBX15* are all distributed near the origin of their respective chromosomes, *MaBBX07*, *MaBBX16*, *MaBBX17*, *MaBBX18*, and *MaBBX20* in the middle of its chromosomes, while *MaBBX03*, *MaBBX09*, *MaBBX10*, *MaBBX14*, and *MaBBX19* are distributed near the ends of their respective chromosomes.

**Figure 2:**
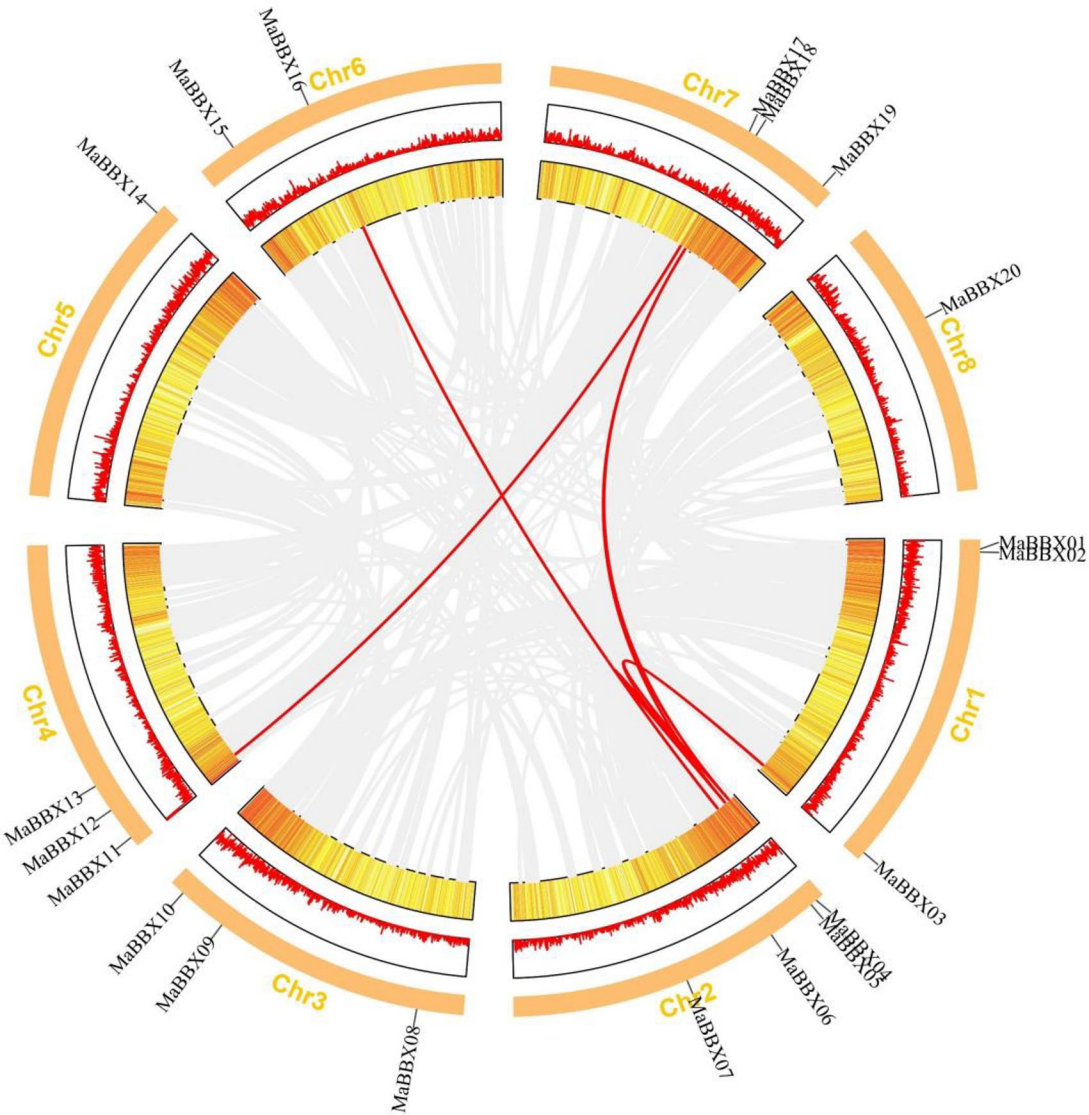
Distribution and synteny analysis of *MaBBX* genes on *Melilotus albus* chromosomes. The approximate chromosomal locations of the *BBX* genes are indicated on the periphery. The colored lines linking genes from different chromosomes denote segmental duplication events.

The duplication of individual genes, chromosomal segment, or of the entire genome itself are the major forces during the course of genome evolution in plants (Magadum et al., 2013). On the *M. albus* chromosome, only one tandem duplication cluster (*MaBBX04*/*MaBBX05*) in the *MaBBX* gene family was found (Figure 2). The *M. albus* genome then revealed six pairs of duplicated segments (*MaBBX03/MaBBX04*, *MaBBX03/MaBBX05*, *MaBBX05/MaBBX16*, *MaBBX04/CsaBBX18*, *MaBBX05/MaBBX18*, *and MaBBX11/MaBBX17*) in the *MaBBX* gene family (Figure 2). The findings suggested that segmental duplication events may have had a larger role in the growth of the *MaBBX* gene family in *M. albus* than tandem duplication. In addition, we determined the value of nonsynonymous (Ka) vs. synonymous (Ks) substitution rates (Ka/Ks) for the tandem and segmentally duplicated gene pairs, which can be used as a proxy for a gene’s selection pressure during evolution. Our findings revealed that all of the Ka/Ks values were smaller than 1, implying that the *MaBBX* genes evolved largely under purifying selection (Table 2). Based on the divergence rate of 4.5 × 10^−9^ synonymous mutations per synonymous site year proposed for *M. albus*, we estimated the time of occurrence of duplicating events of the paralogous *BBX* gene pairs. The results show that the newest (*MaBBX11/MaBBX17*) and oldest (*MaBBX04/MaBBX18*) repeats occurred 68 and 117 Mya, respectively (Table 2).

**Table 2:** Duplicated *MaBBX* genes in *M. albus.*

Gene_1	Gene_2	Ka	Ks	Ka_Ks	Duplication Type	T(MAY)	Note
*MaBBX03*	*MaBBX04*	0.42294	na	na	Segmental	-	High Sequence Divergence Value (pS ≥ 0.75)
*MaBBX03*	*MaBBX05*	0.43216	na	na	Segmental	-	
*MaBBX04*	*MaBBX05*	0.54166	na	na	Tandem	-	
*MaBBX05*	*MaBBX16*	0.90185	na	na	Segmental	-	High Sequence Divergence Value (pS ≥ 0.75)
*MaBBX04*	*MaBBX18*	0.28354	1.056756	0.268314	Segmental	117.4173	
*MaBBX05*	*MaBBX18*	0.52166	na	na	Segmental	-	High Sequence Divergence Value (pS ≥ 0.75)
*MaBBX11*	*MaBBX17*	0.16491	0.61447	0.268379	Segmental	68.2744	

T = Ks/2λ; where λ = 4.5 × 10^−9^.

### Phylogenetic analysis of the *MaBBXs* family

**Figure 3:**
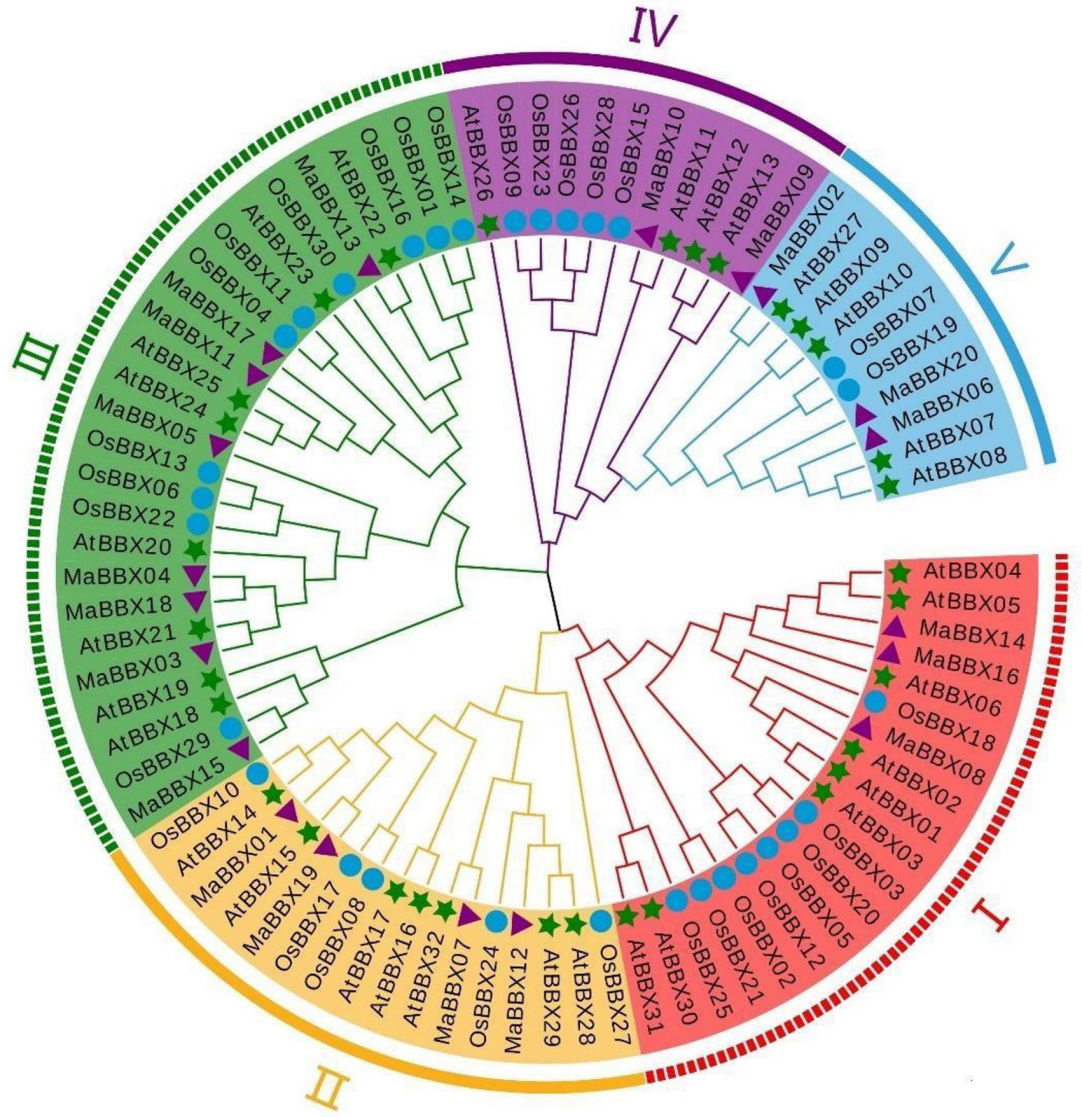
Phylogenetic tree analysis of B-box (BBX) protein family members of *Arabidopsis*, rice, and *Melilotus albus*. *Arabidopsis* At (*Arabidopsis thaliana* L.), rice Os (*Oryza sativa* L.), and *M. albus* Ma (*Melilotus albus*). Five color areas represent five clades; green stars mark *Arabidopsis* BBX protein family members; purple triangles mark *M. albus* BBX protein family members; and blue circle mark rice BBX protein family members. All amino acid sequences used in this analysis are listed in Supplementary Table 1.

We constructed a phylogenetic tree based on 82 protein sequences (Supplementary Table 1) of *BBXs* from three species, including 20 MaBBXs from *M. albus*, 32 MaBBXs from *A. thaliana*, and 30 OsBBXs from *Oryza sativa via* MEGA7 software using the maximum likelihood algorithm. According to the grouping study and analysis of Khanna et al. (2009), we further divided MaBBXs into five subgroups and named as I–V (Figure 3), and the number of members in each subgroup is unevenly distributed. Subfamily III has the most members (8), which are MaBBX03—MaBBX05, MaBBX11, MaBBX13, MaBBX15, MaBBX17, and MaBBX18; the II subfamily has the next largest number of members (4), including MaBBX01, MaBBX07, MaBBX12, and MaBBX19; the I family members include MaBBX08, MaBBX14, and MaBBX16, the V family members are MaBBX02, MaBBX06, and MaBBX20; the IV family has at least two members, MaBBX09 and MaBBX10. In the phylogenetic tree, MaBBX is more closely related to AtBBX, which is consistent with the dicotyledonous plants of *M. albus* and *Arabidopsis*, which are closer to the same ancestor in evolution. The BBX proteins of *M. albus*, *Arabidopsis* and rice were present in all groups, indicating that BBX proteins appeared before the differentiation of monocotyledonous and dicotyledonous plants.

### Gene structure, conserved domains and motifs analysis

**Figure 4:**
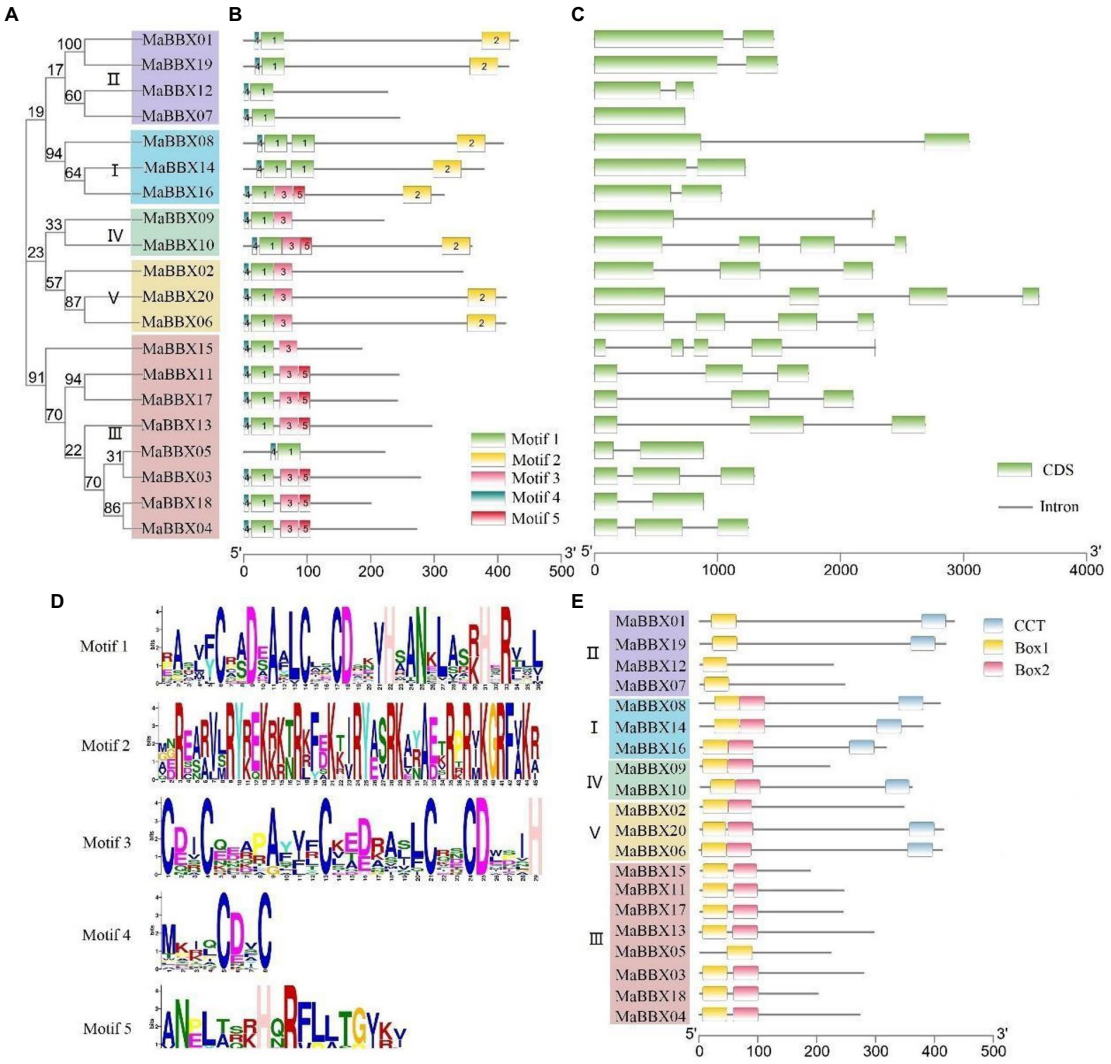
Phylogenetic relationships, gene structures, motifs, and domain organizations of *MaBBXs* in *Melilotus albus*. **(A)** Phylogenetic tree of MaBBX proteins. The assorted colors indicated different clades of *MaBBXs*. **(B)** Distribution of conserved motifs in MaBBX proteins. The scale bar at the bottom indicates the protein lengths, and sequence logos for each conserved motif are shown on the right. **(C)** Intron-exon structures of *MaBBXs*. **(D)** The sequence logo of five conserved motifs. **(E)** The architecture of conserved domains. B-box domain 1, B-box domain 2, and CCT domain are exhibited in different colored boxes.

To gain insight into the structural features of *MaBBX* genes in *M. albus*, we compared their genomic DNA sequences to determine the number of introns and exons within each gene. It was observed that the structures of exons and introns of the *MaBBX* gene of *M. albus* were different among different subfamilies but were conserved within the same subfamily. As shown in Figure 4C, the number and length of introns varied significantly among the different subfamilies. *MaBBX07* has no intron, and all other I and II family members have two exons and one intron. In family III, *MaBBX15* has five exons and four introns, *MaBBX05* and *MaBBX18* have two exons and one intron, and other members have three exons and two introns; In families IV and V, *MaBBX09* has two exons and one intron, *MaBBX02* has three exons and two introns, and the other members have four exons and three introns.

Protein motifs can often predict the function of proteins, and it is also one of the commonly used methods for protein analysis. Motifs analysis of 20 MaBBX protein members showed that the family members contain five motifs, most of which are distributed in the N-terminus (Figure 4B), and only Motif2 is distributed in the C-terminus of the protein, suggesting that this family the N-terminus of proteins may have important biological functions. At the same time, it was observed that each member contains Motif1 and Motif4, and Motif4 is in the front, Motif1 is in the back, and there is no duplication. Except all members of family II, MaBBX08 and MaBBX14 of family I, and MaBBX05 of family III do not contain Motif3, other family members contain Motif3. Box domains are all distributed at the N-terminus, and only a fewer CCT domains were distributed directly at the C-terminus (Figure 4E), which is consistent with the results in Figure 4B. Members of subfamilies I, II, and III are MaBBXs that contain both a B-box domain and a CCT domain. The members of subfamily I contain two Box domains and one CCT domain. In subfamily II, MaBBX01 and MaBBX19 contain one B-box domain and one CCT domain, and MaBBX07 and MaBBX12 contain only one Box domain. Members of subfamilies IV and V contain two Box domains and 0–1 CCT domains. Members of subfamily III do not have a CCT domain but have two and one Box domains, respectively.

### *cis*-acting elements analysis in the promoter regions, MaBBXs protein–protein interaction networks, and prediction of protein tertiary structure of MaBBX

**Figure 5:**
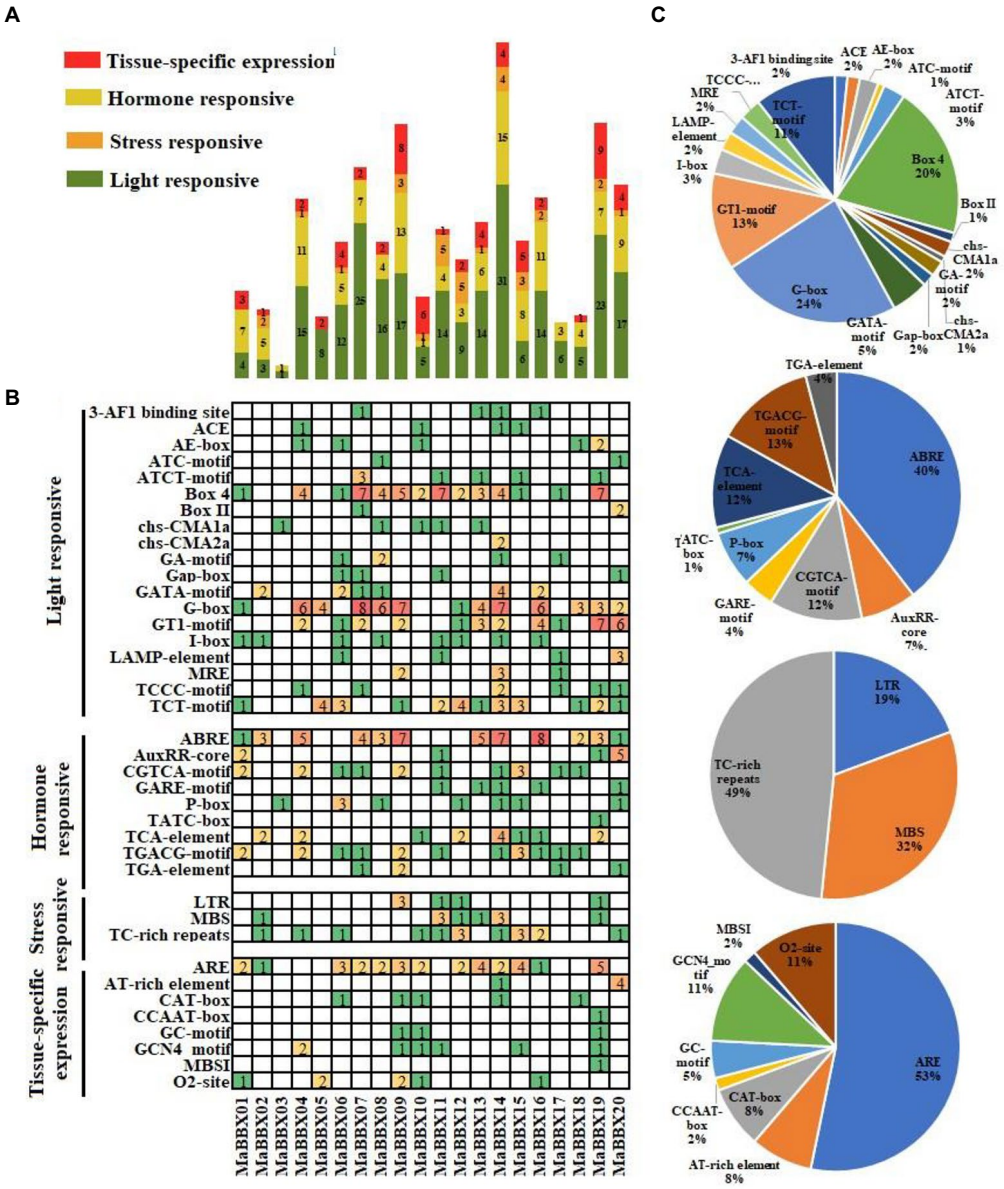
Investigation of *cis*-acting elements in all *MaBBXs*. **(A)** Four categories of *cis-*acting elements in the *MaBBXs*. Different numbers and color of the gird representing the numbers of different elements in these *MaBBXs*. **(B)** Histogram of the *cis*-acting elements in every gene. The different colored histogram indicating the total number of the *cis*-acting elements of each category. **(C)** The ratio of each promoter element in each category.

*cis*-acting elements regulatory sequences located in promoter could control transcriptional expression of corresponding genes to response the internal or environmental signals (Singh et al., 2002). The *cis*-acting elements of *MaBBXs* were divided into four categories: light responsive, tissue-specific expression, stress and hormone responsive (Figures 5A,B). The most frequent occurrences are the light signal response elements, including G-Box, Box 4, TCT-motif, GT1-motif, GATA-motif, AE-box, ATCT-motif, I-box, MRE, and other *cis*-acting elements, the number ranging from 1 to 31. In the hormone responsive category, the ABRE for ABA responsive were the most common *cis*-acting elements, appeared 53 times in 20 *MaBBXs*, accounting for 40% of the hormone responsive *cis*-acting elements (Figure 5C). The others were P-box，GARE motif, and TATC-box for gibberellin-responsive elements, TCA-element for salicylic acid responsive, TGACG-motif and CGTCA-motif for MeJA-responsive, and AuxRR-core and TGA-element for auxin-responsive, which suggested that *MaBBXs* might be regulated by various hormones (Figure 5C). There are three stress response elements in the promoter region of each member of the *MaBBXs*, low temperature response element (LTR), drought stress response element (MBS), and defense stress related element (TC_rich repeats). The types of stress elements contained in the promoter region of each *MaBBX* gene are different, indicating that different members of the *BBX* gene family of *M. albus* respond differently to different stresses.

**Figure 6:**
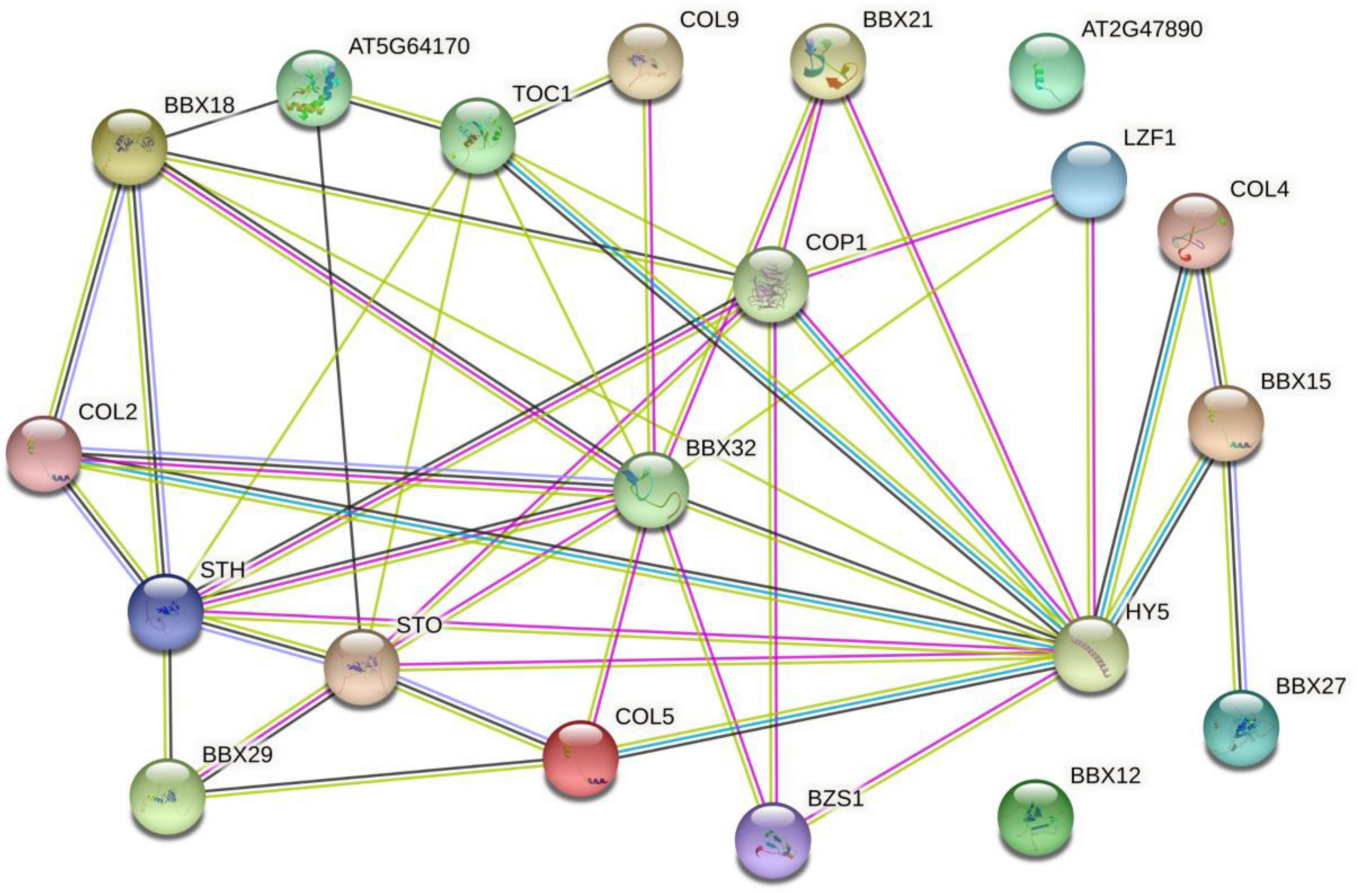
The prediction of the interaction network of MaBBX proteins based on the interactions of their orthologs in *Arabidopsis*. Colored lines between the proteins indicate the various types of interaction evidence. Protein nodes which are enlarged indicate the availability of 3D protein structure information.

The functional relationships among putative BBX proteins were analyzed and predicted using STRING protein interaction database. The network status analyses showed that the expected number of edges and the average local clustering coefficient was 46 and 0.557, respectively. The Protein–protein interaction enrichment value of *p* was <1.0e−16. Many MaBBX proteins potentially interacted with each other, with exceptions of MaBBX09 and MaBBX10 (Figure 6). Besides, other partners of MaBBXs that were also identified included Salt tolerance homologue (*STH*), B-box type zinc finger family protein (BBX18, BBX29, BBX27, BZS1, and STO), Zinc finger protein CONSTANS-LIKE 9 (COL9), Zinc finger protein CONSTANS-LIKE 5 (COL5), Zinc finger protein CONSTANS-LIKE 4 (COL4), Zinc finger protein CONSTANS-LIKE 2 (COL2), B-box type zinc finger protein with CCT domain (AT2G47890, BBX15, and BBX12; Supplementary Table 7). Most of these patterns were involved in seedling photomorphogenesis. For example, STO is a negative regulator of photomorphogenetic UV-B responses by interacting with COP1 and HY5. BZS1 is a positive regulator of seedling photomorphogenesis. Plays a negative role in brassinosteroid responses. BBX21 is a transcriptional activator of a positive regulator of seedling photomorphogenesis. There are also modes (BBX29, AT2G47890 BBX29, COL2, and COL9) take parts in flowering. The remaining patterns (COL5, BBX27, COL4, BBX12, and BBX15) involved in regulation of transcription.

**Figure 7:**
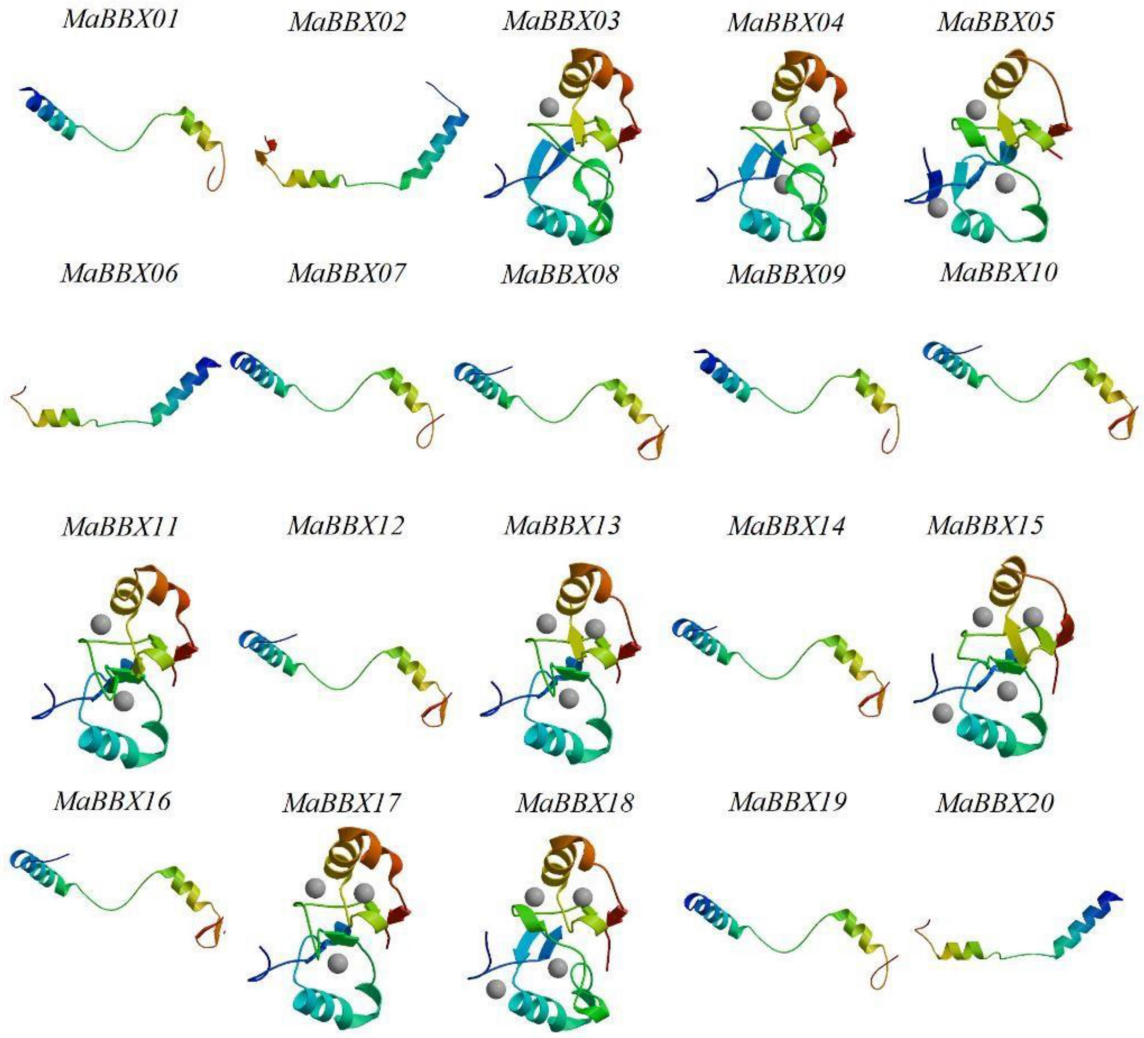
The tertiary structure modeling of MaBBX proteins. The structure image was generated using the SWISS-MODEL software. The validation of the models obtained in this study was also carried out using Ramachandran plot (Supplementary File 1).

The tertiary structure of a protein refers to the non-geometrically further folding or coiling of the entire polypeptide chain on the basis of the secondary structure to form a complex spatial structure, which is mainly maintained by hydrogen bonds, van der Waals forces, hydrophobic interactions and electrostatic interactions. In this paper, the SWISS-MODEL software was used to construct the tertiary structure of the target protein by homology modeling. At the same time, we used Ramachandran Plot to evaluate the rationality of the protein tertiary structure model. The evaluation results showed that the amino acid residues in the acceptable region of all MaBBXs protein models were greater than 90% (Supplementary File 1), so we believed that the protein structure was reasonable. The MaBBX protein of *M. albus* contains spatial conformations such as α-helix, βsheet, β-turn, and random coil (Figure 7). The overall structural similarity is different, and the complexity is average. The tertiary structures of members of the same subgroup of MaBBX proteins are highly similar, indicating that the protein structure is related to species evolution.

### qRT-PCR analysis of *MaBBXs* under different abiotic stress

**Figure 8:**
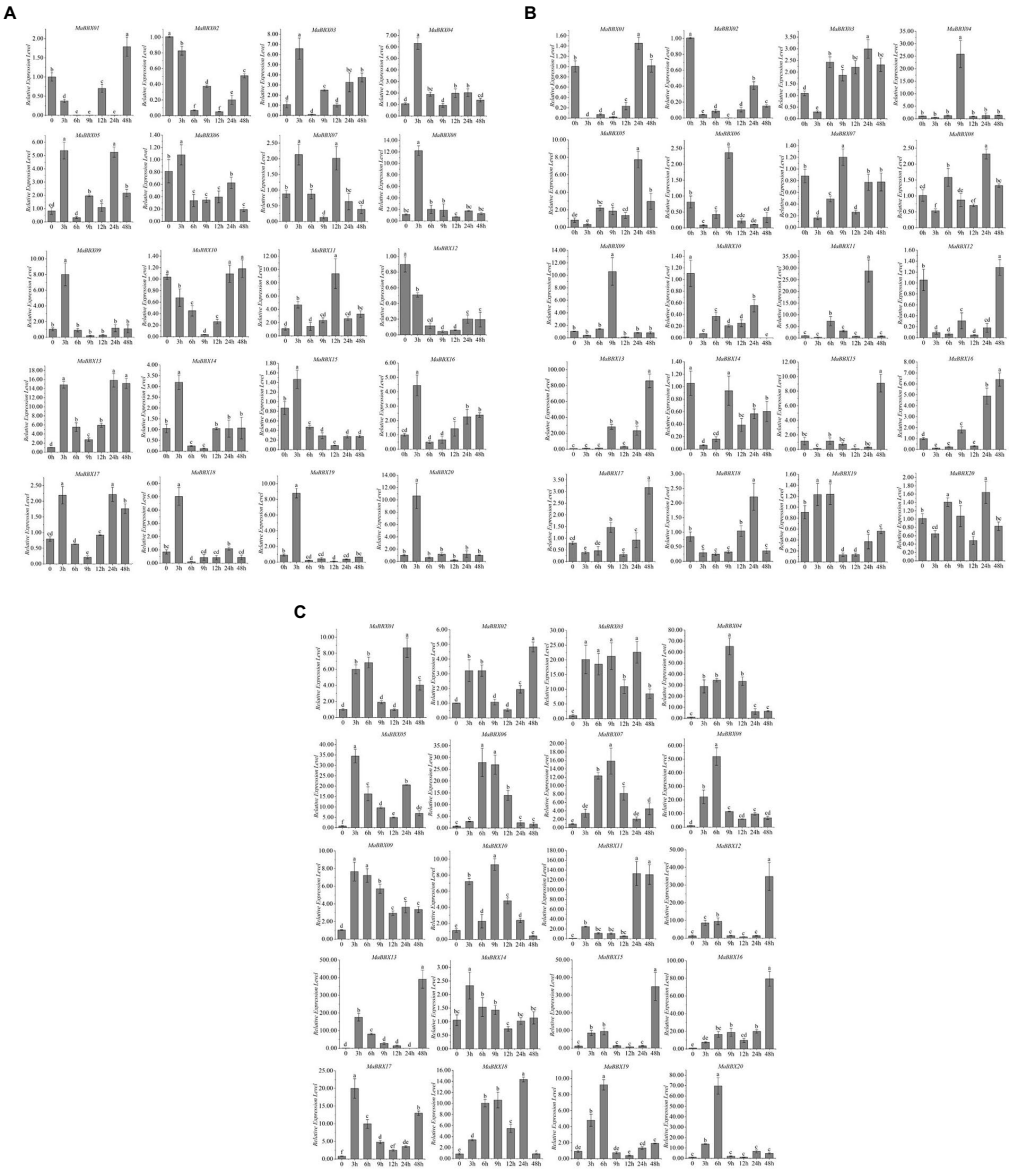
Expression profiles of the *MaBBXs* under abiotic stresses. The expression patterns of *BBX* genes using qRT-PCR in leaf under salt **(A)**, cold **(B)**, and dark **(C)** treatments at 0, 3, 6, 9, 12, 24, and 48 h of treatment, respectively. β-Tubulin was used as internal control. The *y*-axis exhibits the relative expression level. The error bars mean the SDs of three biological replicates. Lowercase letters represented significance at 0.05 level (*p* < 0.05).

To further explore *MaBBX* gene expression patterns under abiotic stresses and identify genes important for improving tolerance to abiotic stresses, *M. albus* were subjected to abiotic stresses such as cold, salt, and dark. A total of 20 *MaBBXs* were performed quantitative real-time RT-PCR at different time points (0, 3, 6, 9,12, 24, and 48 h) after abiotic treatments, and the expression levels of these genes are listed in Supplementary Table 2. In general, all *MaBBX* genes in *M. albus* showed different expression patterns, and the expression of these 20 *MaBBX* genes could be induced by all the above treatments. Compared with the control, 16 genes showed higher expression levels after salt treatment for 3 h, and the expression levels of four genes (*MaBBX01*, *MaBBX02*, *MaBBX10*, and *MaBBX12*) were lower, and the expression levels of all genes decreased after 6 h of treatment (Figure 8A). *MaBBX07* and *MaBBX11* were significantly upregulated at 12 h, *MaBBX05*, *MaBBX10*, *MaBBX13*, and *MaBBX17* were upregulated at 24 h, and *MaBBX01* were significantly upregulated at 48 h. The expression levels of 13 genes (*MaBBX 02*-*04*, *MaBBX06*, *MaBBX08*–*09*, *MaBBX12*, *MaBBX14*-*16*, and *MaBBX18*-*20*) were still low in the following four time points (9, 12, 24, and 48 h). Under cold stress condition, 20 *MaBBX* genes showed different expression levels (Figure 8B). The expression levels of 10 genes (*MaBBX3*-*4*, *MaBBX8*-*9*, *MaBBX14-16*, and *MaBBX18*-*20*) were significantly upregulated at cold treatment for 3 h, and the expression levels at the following five time points were all at lower levels compared with the control. *MaBBX02*, *MaBBX10*, and *MaBBX12* expression down-regulated under cold treatment. The expression levels of *MaBBX01* and *MaBBX17* were stable or downregulated within 24 h after treatment, but upregulated at 48 h, suggesting that some *BBXs* may be induced under severe stress conditions. For dark stress, compared with the control, the expression levels of all 20 genes were upregulated at 3 h of treatment (Figure 8C). Taken together, we found the expression patterns of some *MaBBX* genes are similar under salt, cold, and dark treatments (Figure 8). In addition, our results suggested that the expression levels of *MaBBX13* were induced by salt, cold, and dark stresses, suggesting that the genes might be important candidates for improving tolerance to abiotic stresses.

### Subcellular location of MaBBXs

**Figure 9:**
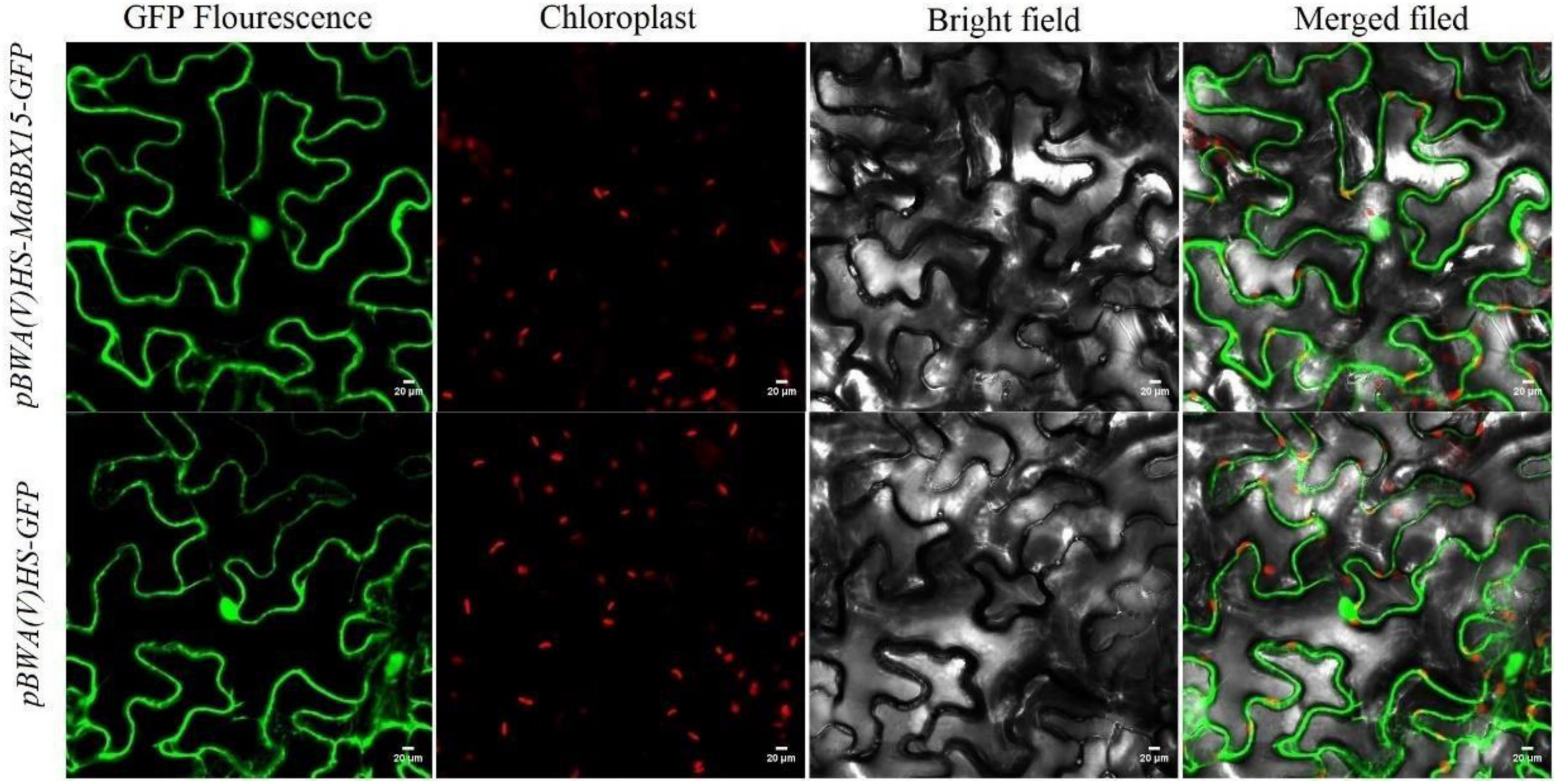
Subcellular localization of the GFP-fused MaBBX15 protein. The fusion protein pBWA(V)HS-MaBBX15-GFP and the control vector were transiently expressed in tobacco leaves and then observed by fluorescence microscopy. Scale bar was 20 μm.

The results of protein subcellular localization indicated that MaBBXs were likely to be distributed in the nucleus, cytoplasm, and mitochondrial matrix of *M. albus* (Table 1). MaBBX15 which display organ-specific expression patterns or are highly responsive to abiotic stresses were selected to verify their cellular location using transient expression assay. The green fluorescence signals from pBWA(V)HS-MaBBX15-GFP fusion proteins were all observed specifically in the nucleus and cytoplasm of tobacco leaves, suggesting that the fusion proteins were localized in the nucleus and cytoplasm (Figure 9), and these were consistent with the prediction results (Table 1).

## Discussion

B-boxes play a vital role in regulating plant flowering (Nagaoka and Takano, 2003) and responding to plant biotic (Vaishak et al., 2019) and abiotic stresses (Ledger et al., 2001; Zhao et al., 2021). The function and evolution of BBX genes have been identified in *Arabidopsis* (Wang et al., 2014), *Oryza sativa* (Bai et al., 2016), *Solanum lycopersicum* (Chu et al., 2016), *Malus domestica* (Liu et al., 2018), *Vitis vinifera* (Bai et al., 2019), *Pyrus pyrifolia* (Bai et al., 2019), *Chrysanthemum* (Xu et al., 2020), *Brassica rapa* (Singh et al., 2021), and *Cucumis sativus* (Obel et al., 2022). Although the *M. albus* full genome has been sequenced, the BBX gene family is yet to be identified by a genome-wide study in *M. albus*. This is the first time that bioinformatics technologies have been used to identify and define the BBX gene family in *M. albus*.

In this study, a total of 20 *MaBBXs* genes were identified at the *M. albus* genome level, and the obtained 20 *MaBBXs* genes were divided into five subfamilies. The same subfamily has similar exon-intron numbers and conserved motifs, but the gene structures and conserved motifs of different subfamily members are quite different (Figure 4). Genes in the same subfamily are speculated to have similar biological functions. The five *BBX* subfamily genes are distributed in both monocotyledonous and dicotyledonous plants, indicating that the differentiation among *BBX* family members precedes species differentiation. Generally, gene duplication events exist in gene families such as in MaNACs and MaMYBs, there are 48 and 136 duplicated gene pairs (Chen et al., 2021a). In *M. albus*, one tandem duplication and six segmental duplications were discovered in the *MaBBX* genes (Figure 2), implying that the *BBX* genes have expanded to a vast extent. Previous studies of *BBX* in pear (*Pyrus communis*), sweet potato (*Ipomoea batatas*), grapevine (*Vitis vinifera*), and tomato (*Solanum lycopersicum*) all reported that they were predominantly located in the nucleus (Chu et al., 2016; Cao et al., 2017; Wei et al., 2020; Hou et al., 2021). However, we found that BBX is not only located in the nucleus but also in the cytoplasm in *M. albus*. This finding may have important implications for the study of BBX function.

The protein interaction network can link the unknown functional protein MaBBXs to the known functional *Arabidopsis* proteins, which will help to further understand the biological function of the protein. Protein network interactions suggest that most of the proteins in MaBBXs may act as regulators of seedling photomorphogenesis and flowering in *M. albus*. Previous studies have found that gene family members located in the same subgroup have a common origin and conserved functions, and the functions of orthologous or paralogous genes can be determined by the functions of known genes (Nakashima et al., 2009). Studies have shown that *AtBBX21* coordinates with HY5 and ABI5 on the ABI5 promoter, and that these transcriptional regulators work together to integrate light and ABA signaling in *Arabidopsis* (Xu et al., 2016). In this study, *MaBBX03* is orthologous to *AtBBX21*, which may have similar functions to *AtBBX21* and participate in ABA signaling. *AtBBX22* plays a positive regulatory role in the de-etiolation process of *Arabidopsis* and participates in photomorphogenesis by regulating the synthesis of brassinolide (Fan et al., 2012). *MaBBX13* and *AtBBX22* are orthologous genes, and it is speculation that this gene may regulate photomorphogenesis through the synthesis of brassinolide. Overexpression of *AtBBX20* increases the expression of HY5 and promotes photomorphogenesis. At the same time, HY5 can also interact with *AtBBX20*, but negatively regulates the expression of *AtBBX20*, forming a negative feedback regulation (Wei et al., 2016). *AtBBX28* negatively regulates photomorphogenesis by forming a non-DNA-binding heterodimer with HY5 that prevents HY5 from binding to its downstream target genes (Lin et al., 2018). Therefore, we speculate that *MaBBX04* and *MaBBX12*, which are homologous to *AtBBX20* and *AtBBX28*, may be involved in plant photomorphogenesis. *AtBBX19* can interact with plant circadian clock system element (PRR) to coordinate the circadian rhythm in *Arabidopsis*, and this transcriptional regulator plays an indispensable role in regulating plant circadian rhythm (Yuan et al., 2021), we speculate that it has the same function as *AtBBX19*. Derived *MaBBX15* may be a regulator of *M. albus* circadian rhythm.

*B-box* gene family plays a very important role in plant growth and development (Wang et al., 2013b; Xiao et al., 2019). In this study, *MaBBXs* found that in addition to the light-responsive elements G-Box, Box 4, TCT-motif, etc., the low-temperature-responsive element LTR, the stress-responsive element TC_rich repeats, and the drought-induced *MYB* transcription factor binding site MBS were also found on *MaBBXs*. This indicated that the *BBX* family genes of *M. albus* were not only related to photomorphogenesis but also involved in the response to adversity stress. It has been previously reported that *AtBBX24* protein can bind to the promoter region of a *HPPBF-1* gene and promote its expression, which was induced by salt stress, indicating that *AtBBX24* was indirectly involved in the molecular pathway related to increased plant salt tolerance (Nagaoka and Takano, 2003). A considerable part of *BBX* genes in apple (*Malus domestica*) was upregulated under high salt and low-temperature treatment. The *CmBBX24* and *CmBBX22* genes of *Chrysanthemum* are also involved in the plant’s response to low temperature, etc. (Yang et al., 2014b). High salt treatment can induce the expression of *SsBBX24* gene in potatoes (*Solanum tuberosum*), and the length of sunshine can also regulate the response of *SsBBX24* to salt stress (Kiełbowicz-Matuk et al., 2014). In addition, dark treatment can induce the expression of wheat *TaBBX2.15* and *TaBBX2.23* (Chen et al., 2021b). Therefore, the relative expression levels of *MaBBXs* under low temperature, dark, and salt treatments were further analyzed, and the results showed that most of *MaBBXs* were involved in the response to low temperature, dark, and salt stress of *M. albus*. In addition, plant *BBX* participates in hormone-mediated stress responses. Mutation of *AtBBX21* in *A. thaliana* reduces plant sensitivity to ABA and the rate of water loss, thereby improving plant drought resistance (Imtiaz et al., 2015). The promoter regions of the 20 *MaBBXs* in this study also contain *cis*-acting elements that respond to five plant hormones, abscisic acid (ABA), methyle jasmonate (MeJA), gibberellin (GA), auxin, and salicylic acid (SA). Their involvement in hormone-mediated responses to adversity stress requires further study.

## Conclusion

In this study, a total of 20 *BBX* homologous genes were identified in the *M. albus* genome. Phylogenetic and evolutionary relationships, gene structure analysis, and motif analysis indicated the conservation of *MaBBX* families and that some members diverged from their ancestors. The current study reported that all the promoters of *MaBBXs* contained hormone and stress-related *cis*-elements. The transcription of certain *MaBBXs* can be induced or repressed under abiotic stresses (salt, cold, and dark), indicating that *MaBBX* genes may have essential functions in various biological processes. Taken together, these results lay a solid foundation for further research on the function of the *BBX* family genes of the *M. albus*. However, functional studies of *MaBBXs* in *M. albus* require further validation. In addition, the mode of the response of these genes to biotic stresses such as hormone treatment is unclear and requires further study.

## Data availability statement

Publicly available datasets were analyzed in this study. This data can be found at: NCBI, PRJNA674670.

## Author contributions

XuL, YY, and XZha conceived and designed the experiments. XiL, XaL, XZhu, and FH performed the experiments. BM, ZM, and ZX analyzed the data. LN wrote the paper. All authors contributed to the article and approved the submitted version.

## Funding

This research was financially supported by The Scientific Research Start-up Funds for Openly Recruited Doctors of Gansu Agriculture University (2017RCZX32), Research on the Coordinated Relationship between Land Urbanization and Population Cities (GSAU-ZL-2015-046), and National Natural Science Foundation of China (31601984) availability of data and materials.

## Conflict of interest

The authors declare that the research was conducted in the absence of any commercial or financial relationships that could be construed as a potential conflict of interest.

## Publisher’s note

All claims expressed in this article are solely those of the authors and do not necessarily represent those of their affiliated organizations, or those of the publisher, the editors and the reviewers. Any product that may be evaluated in this article, or claim that may be made by its manufacturer, is not guaranteed or endorsed by the publisher.

## Footnotes

^1^ http://psort1.hgc.jp/form.html

^2^ http://www.csbio.sjtu.edu.cn/bioinf/Cell-PLoc-2/

^3^ https://itol.embl.de

^4^ http://www.ebi.ac.uk/thornton-srv/databases/pdbsum/Generate.html
