# Supplementary material for: Characterization of B-box family genes and their expression profiles under abiotic stresses in the Melilotus albus
Source: Front Plant Sci. 2022 Sep 29;13:990929. doi: 10.3389/fpls.2022.990929 (PMC9559383; doi:10.3389/fpls.2022.990929)
Supplement: Supplementary file 7 [file Table_7.docx]

**Supplementary Table7. The functional relationships between MaBBX proteins and known *Arabidopsis* proteins.**

| proteins in *Arabidopsis thaliana* | proteins in *M. albus* | Functional annotation |
| --- | --- | --- |
| COL9 | MaBBX20 | Zinc finger protein CONSTANS-LIKE 9; This gene belongs to the CO (CONSTANS) gene family. This gene family is divided in three subgroups: groups III, to which COL9 belongs, is characterized by one B-box (supposed to regulate protein-protein interactions) and a second diverged zinc finger. COL9 downregulates expression of CO (CONSTANS) as well as FT and SOC1 which are known regulatory targets of CO. |
|  | MaBBX06 |  |
| BBX27 | MaBBX02 | B-box zinc finger family protein; Its function is described as sequence-specific DNA binding transcription factor activity, zinc ion binding; Involved in regulation of transcription; Located in endomembrane system, intracellular; Expressed in 14 plant structures; Expressed during 8 growth stages; Contains the following InterPro domains: Zinc finger, B-box (InterPro:IPR000315); BEST Arabidopsis thaliana protein match is: CONSTANS-like 9 (TAIR:AT3G07650.4); Has 1632 Blast hits to 1343 proteins in 109 species: Archae - 0; Bacteria - 2; Metazoa - 1; Fungi - 0; Plants - 1580; Viruses - 0; |
| COL5 | MaBBX14 | Zinc finger protein CONSTANS-LIKE 5; CONSTANS-like 5 (COL5); Its function is described as sequence-specific DNA binding transcription factor activity, zinc ion binding; Involved in regulation of transcription; Located in intracellular; Expressed in 23 plant structures; Expressed during 13 growth stages; Contains the following InterPro domains: CCT domain (InterPro:IPR010402), Zinc finger, B-box (InterPro:IPR000315); BEST Arabidopsis thaliana protein match is: CONSTANS-like 4 (TAIR:AT5G24930.1); Has 30201 Blast hits to 17322 proteins in 780 species: Archae - 12; Bacteria - 1396; Metazoa |
| COL4 | MaBBX16 | Zinc finger protein CONSTANS-LIKE 4; CONSTANS-like 4 (COL4); Its function is described as sequence-specific DNA binding transcription factor activity, zinc ion binding; Involved in regulation of transcription; Located in intracellular, chloroplast; Expressed in 21 plant structures; Expressed during 13 growth stages; Contains the following InterPro domains: CCT domain (InterPro:IPR010402), Zinc finger, B-box (InterPro:IPR000315); BEST Arabidopsis thaliana protein match is: CONSTANS-like 3 (TAIR:AT2G24790.1); Has 3148 Blast hits to 2480 proteins in 140 species: Archae - 0; Bacteria - 4; |
| BBX21 | MaBBX03 | Long hypocotyl under shade; Transcription activator that acts as positive regulator of seedling photomorphogenesis. Acts downstream of COP1 and play an important role in early and long-term adjustment of the shade avoidance syndrome (SAS) responses in natural environments |
|  | MaBBX05 |  |
| BBX18 | MaBBX15 | B-box zinc finger family protein; Acts as negative regulator of seedling photomorphogenesis. Acts as a negative regulator of blue light-mediated inhibition of hypocotyl elongation through increase of bioactive gibberellin levels. Acts as a repressor of thermotolerance by modulating expression of a set of heat shock- responsive genes |
| BBX15 | MaBBX01 | B-box type zinc finger protein with CCT domain; Its function is described as sequence-specific DNA binding transcription factor activity, zinc ion binding; Involved in regulation of transcription; Located in membrane; Expressed in leaf; Contains the following InterPro domains: CCT domain (InterPro:IPR010402), Zinc finger, B-box (InterPro:IPR000315); BEST Arabidopsis thaliana protein match is: B-box type zinc finger protein with CCT domain (TAIR:AT1G68520.1); Has 3476 Blast hits to 2333 proteins in 129 species: Archae - 0; Bacteria - 2; Metazoa - 0; Fungi - 0; Plants - 3380; Viruses - 0 |
|  | MaBBX19 |  |
| BBX12 | MaBBX09 | B-box type zinc finger protein with CCT domain; Its function is described as sequence-specific DNA binding transcription factor activity, zinc ion binding; Involved in regulation of transcription; Located in membrane; Expressed in leaf; Contains the following InterPro domains: CCT domain (InterPro:IPR010402), Zinc finger, B-box (InterPro:IPR000315); BEST Arabidopsis thaliana protein match is: B-box type zinc finger protein with CCT domain (TAIR:AT1G68520.1); Has 3476 Blast hits to 2333 proteins in 129 species: Archae - 0; Bacteria - 2; Metazoa - 0; Fungi - 0; Plants - 3380; Viruses - 0 |
| BZS1 | MaBBX18 | B-box zinc finger family protein; Acts as positive regulator of seedling photomorphogenesis. Plays a negative role in brassinosteroid responses |
|  | MaBBX04 |  |
| COL2 | MaBBX08 | Zinc finger protein CONSTANS-LIKE 2; Homologous to the flowering-time gene CONSTANS (CO) encoding zinc-finger proteins |
| STH | MaBBX11 | Salt tolerance homologue; Acts as negative regulator of seedling photomorphogenesis. BBX25/STH and BBX24/STO function as transcriptional corepressors of HY5 activity, leading to the down-regulation of BBX22 expression. BBX25/STH acts additively with BBX24/STO during de- etiolation and the hypocotyl shade avoidance response |
| BBX29 | MaBBX12 | B-box type zinc finger family protein; Its function is described as sequence-specific DNA binding transcription factor activity, zinc ion binding; Involved in response to cold, regulation of transcription; Located in intracellular; Expressed in 11 plant structures; Expressed during 4 anthesis, F mature embryo stage, petal differentiation and expansion stage, E expanded cotyledon stage, D bilateral stage; Contains the following InterPro domains: Zinc finger, B-box (InterPro:IPR000315); BEST Arabidopsis thaliana protein match is: B-box type zinc finger family protein (TAIR:AT4G27310.1); |
|  | MaBBX07 |  |
| LZF1 | MaBBX13 | annotation not available |
| STO | MaBBX17 | B-box zinc finger family protein; Acts as negative regulator of seedling photomorphogenesis and light-regulated inhibition of hypocotyl elongation BBX24/STO and BBX25/STH function as transcriptional corepressors of HY5 activity, leading to the down- regulation of BBX22 expression. BBX24/STO acts additively with BBX25/STH during de-etiolation and the hypocotyl shade avoidance response. Functions as negative regulator of photomorphogenic UV-B responses by interacting with both COP1 and HY5. May act as a transcription factor in the salt-stress response |
| AT2G47890 | MaBBX10 | B-box type zinc finger protein with CCT domain; Its function is described as sequence-specific DNA binding transcription factor activity, zinc ion binding; Involved in regulation of transcription; Located in intracellular; Expressed in stem, inflorescence meristem, root, flower, stamen; Expressed during 4 anthesis, petal differentiation and expansion stage; Contains the following InterPro domains: CCT domain (InterPro:IPR010402), Zinc finger, B-box (InterPro:IPR000315); BEST Arabidopsis thaliana protein match is: B-box type zinc finger protein with CCT domain (TAIR:AT1G28050.1); Has 32 |
