# Supplementary material for: Characterization of B-box family genes and their expression profiles under abiotic stresses in the Melilotus albus
Source: Front Plant Sci. 2022 Sep 29;13:990929. doi: 10.3389/fpls.2022.990929 (PMC9559383; doi:10.3389/fpls.2022.990929)
Supplement: Supplementary file 8 [file Data_Sheet_1.ZIP › Additional file2/MaBBX02.pdf]

PROCHECK

Ramachandran Plot

saves

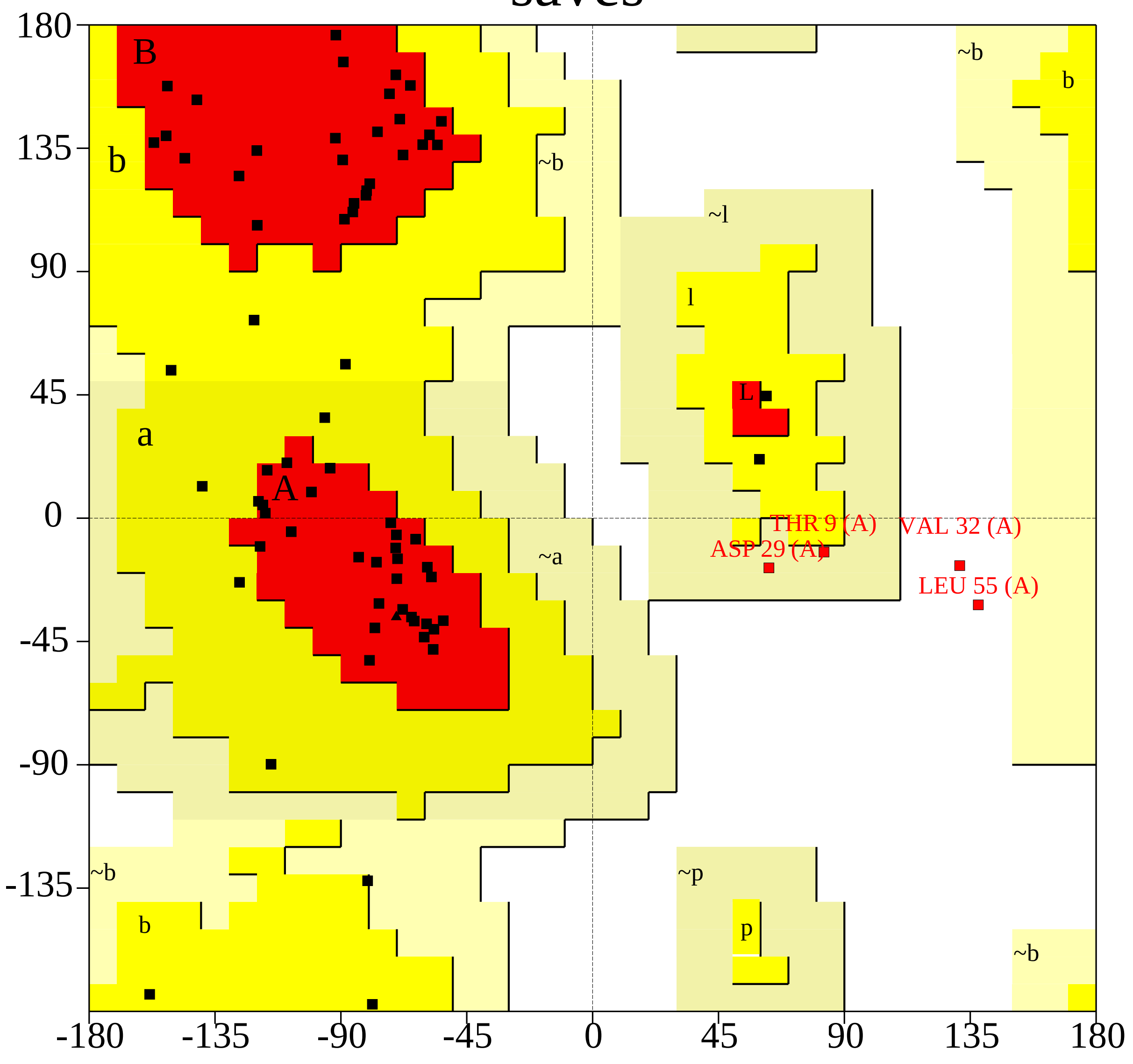

Plot statistics

Residues in most favoured regions [A,B,L]	66	90.4%
Residues in additional allowed regions [a,b,l,p]	7	9.6%
Residues in generously allowed regions [~a,~b,~l,~p]	2	2.7%
Residues in disallowed regions	2	2.7%

Number of non-glycine and non-proline residues	73	100.0%
Number of end-residues (excl. Gly and Pro)	2	
Number of glycine residues (shown as triangles)	1	
Number of proline residues	1	

Total number of residues	77	

Based on an analysis of 118 structures of resolution of at least 2.0 Angstroms and R-factor no greater than 20%, a good quality model would be expected to have over 90% in the most favoured regions.
