# Supplementary material for: Characterization of B-box family genes and their expression profiles under abiotic stresses in the Melilotus albus
Source: Front Plant Sci. 2022 Sep 29;13:990929. doi: 10.3389/fpls.2022.990929 (PMC9559383; doi:10.3389/fpls.2022.990929)
Supplement: Supplementary file 8 [file Data_Sheet_1.ZIP › Additional file2/MaBBX20.pdf]

PROCHECK

# Ramachandran Plot

## saves

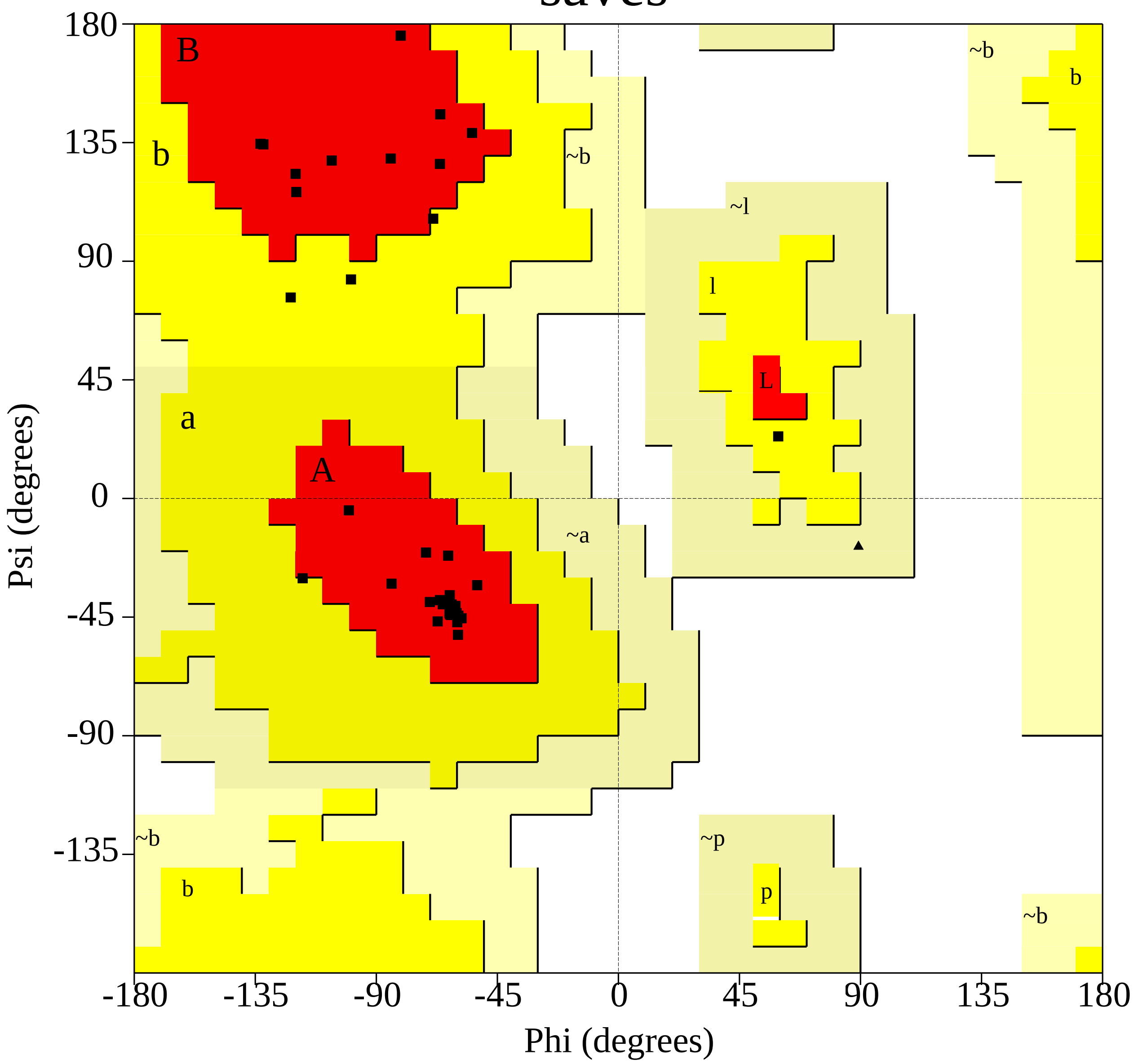


### Plot statistics

| | | |
|---|---|---|
| Residues in most favoured regions [A,B,L] | 36 | 92.3% |
| Residues in additional allowed regions [a,b,l,p] | 3 | 7.7% |
| Residues in generously allowed regions [~a,~b,~l,~p] | 0 | 0.0% |
| Residues in disallowed regions | 0 | 0.0% |
| | ------- | |
| Number of non-glycine and non-proline residues | 39 | 100.0% |
| Number of end-residues (excl. Gly and Pro) | 2 | |
| Number of glycine residues (shown as triangles) | 1 | |
| Number of proline residues | 0 | |
| | ------- | |
| Total number of residues | 42 | |

Based on an analysis of 118 structures of resolution of at least 2.0 Angstroms and R-factor no greater than 20%, a good quality model would be expected to have over 90% in the most favoured regions.
